# Research Progress in the Mechanism of Effect of PRP in Bone Deficiency Healing

**DOI:** 10.1155/2013/134582

**Published:** 2013-04-04

**Authors:** Ning Zhang, Yong-Ping Wu, Sheng-Jun Qian, Chong Teng, Shuai Chen, Hang Li

**Affiliations:** Department of Orthopaedics, The Second Affiliated Hospital, School of Medicine, Zhejiang University, 88 Jiefang Road, Hangzhou 310009, China

## Abstract

Platelet-rich plasma (PRP) therapy is a recently developed technique that uses a concentrated portion of autologous blood to try to improve and accelerate the healing of various tissues. There is a considerable interest in using these PRP products for the treatment used in bone deficiency healing. Because PRP products are safe and easy to prepare and administer, there has been increased attention toward using PRP in numerous clinical settings. The benefits of PRP therapy appear to be promising, and many investigators are exploring the ways in which this therapy can be used in the clinical setting. At present, the molecular mechanisms of bone defect repair studies have focused on three aspects of the inflammatory cytokines, growth factors and angiogenic factors. The role of PRP works mainly through these three aspects of bone repair. The purpose of this paper is to review the current evidence on the mechanism of the effect of PRP in bone deficiency healing.

## 1. Introduction

Bone defects are very challenging in the management of patients. They can result from a high-energy traumatic event, from large bone resection for different pathologies such as tumour or infection, or from the treatment of complex fractures [1]. Significant bone defects or nonunion fractures may usually require bone grafting in order to fill the defect, for bone grafts could fill spaces and provide support, and enhance the biological repair of the defect. Bone grafting is recommended as a common surgical procedure [2].

Autologous bone grafts are widely considered as a gold standard for a number of reasons, including osteogenic, osteoconductive, osteoinductive properties, and the lack of disease transmission or of immunogenicity [3]. They can be utilized to treat patients with nonunion, poor osteogenic potential, highly comminuted fractures and osteomyelitis. However, the use of autograft may be at risk of major drawbacks, such as limited availability and variable quality of the graft, hematoma, infection, increased operative time and bleeding, chronic donor site pain, and additional cost [4]. In addition, the amount of autograft is limited. To overcome these disadvantages, bone substitutes may be used instead [5]. Many recent studies have focused on the development of novel bone graft substitutes for the last decades [4, 6–8].

Platelets play an important role in the initial wound healing, and bleeding from the wound leads to rapid activation of platelets that release multiple growth factors and cytokines involved in healing [9]. Since the first demonstration of new bone formation with a combination of autogenous bone graft and PRP, this pioneering work, a large body of data obtained by preclinical animal studies has supported the utility of PRP in human clinical settings [10, 11]. PRP may provide optimistic prospects for bone graft procedures.

## 2. Platelet-Rich Plasma (PRP)

Platelet-rich plasma (PRP), with a platelet concentration of at least 1 000 000 platelets/L in 5 mL of plasma, containing a 3-to-5-fold increase in growth factor concentrations, is associated with the enhancement of healing [12]. Cellular events that follow tissue damage are controlled among others by platelets and the released growth factors. Platelets release a large variety of growth factors and cytokines after they adhere, aggregate, and form a fibrin mesh [13]. Furthermore, artificial recombinant growth factors often require further synthetic or animal proteins as carriers. PRP in contrast could serve as a natural carrier itself [14].

## 3. Mechanisms of PRP on Repair of Bone Defects

Bone has a substantial capacity for repair and regeneration in response to injury occurs by surgery, various diseases, or trauma. Both processes involve a complex integration of cells, growth factors, and the extracellular matrix [15, 16]. PRP can potentially enhance healing by the delivery of various growth factors and cytokines from the *α*-granules contained in platelets [17]. The basic cytokines, which identified platelets, play important roles in cell proliferation, chemotaxis, cell differentiation, and angiogenesis. Bioactive factors are also contained in the dense granules in platelets. The dense granules contain serotonin, histamine, dopamine, calcium, and adenosine [18]. These nongrowth factors have fundamental effects on the biologic aspects of wound healing. At present, the molecular mechanisms of bone defect repair studies have focused on three aspects of the inflammatory cytokines, growth factors, and angiogenic factors. The role of PRP works through these three aspects of bone repair [19]. Growth factors and cytokines in PRP associated with diferent mechanisms are showed in Table 1. 

### 3.1. PRP in the Role of Inflammatory Cytokines Promotes Bone Repair

There is increasing evidence that inflammation plays a vital role in early fracture repair [20]. Consequently, platelets are stimulated to aggregate and secrete growth factors, cytokines, and hemostatic factors critical in the early stages of the intrinsic and extrinsic pathways of the clotting cascade. Inflammatory reactions involve a number of biochemical and cellular alterations, the extent of which correlates with the extent of the initial trauma [21, 22]. Histamine and serotonin are released by platelets and both function to increase capillary permeability, which allows inflammatory cells greater access to the wound site and activates macrophages [23, 24]. Adenosine receptor activation modulates inflammation during wound healing [25].

 The major proinflammatory cytokines that are responsible for early responses are IL1, IL6, and TNF-alpha [26, 27]. The expression of TNF-*α* and IL-1 in fractures follows a biphasic pattern, with a peak during the initiation of fracture repair, followed by a second peak at the transition from chondrogenesis to osteogenesis during endochondral maturation [28, 29]. A femoral fracture model using IL-6 knockout mice also demonstrated delayed callus remodeling and mineralization [30], and both TNF-*α* and IL-1*β* have been shown to recruit osteoblasts [31]. Furthermore, a study using human-fracture bone fragments and murine model identified the pivotal role of TNF-*α* in enhancing fracture healing and showed that PRP may suppress IL-1 release from activated macrophages [32].

### 3.2. PRP in the Role of Growth Factors Promotes Bone Repair

Autologous platelet preparations have demonstrated the potential to modify the natural healing pathway of bone in several ways. The action is related to the increased concentration of growth factors and bioactive proteins released by activated platelets, which seem able to help the regeneration of tissues that otherwise have low healing potential [33, 34], potentially restoring biomechanical properties similar to normal bone [35]. The application of PRP amplifies the surge of chemical mediators to the microenvironment of the injured area. The growth factors released from the platelets include platelet-derived growth factor (PDGF) [36], transforming growth factor-(TGF-) beta, platelet-derived epidermal growth factor (PDEGF), platelet-derived angiogenesis factor (PDAF) [37], insulin-like growth factor (IGF-1), and platelet factor 4 (PF-4) [36, 38, 39]. The best known growth factors include PDGF, TGF-*β*eta1 and beta2, and IGF-1. Other growth factors present in platelet granules are the vascular endothelial growth factors (VEGF) and endothelial growth factors (EGF) [40, 41]. Each of these factors has its own role to play. 

#### 3.2.1. PDGF

PDGF could be found in platelets and especially in the alpha granules and could also be found in other cells, such as macrophages, endothelial cells, monocytes, and fibroblasts, as well as in bone matrix [42]. It has a heterodimer structure, consisting of two different A and B chains. AA and BB homodimers are also found in platelets and demonstrate similar activity [43]. The reason for three distinct forms remains unclear, but differential binding by various receptor cells such as endothelium, fibroblasts, macrophages, and marrow stem cells has been suggested [44]. As a result of the presence of platelets in the blood clot, PDGF is the first growth factor in a wound, stimulating revascularization, collagen synthesis, and bone regeneration [45, 46]. PDGF's role in the wound healing process consists in the stimulation of mitogenesis to increase the number of regenerative cells, angiogenesis stimulation to support the development of new vessels, and the activation of macrophages responsible for wound cleaning and being a secondary source of growth factors [47]. The PDGF acts through alpha receptors from cell membrane, releasing a cascade of intracellular reactions triggering expression of appropriate genes. Damage to PDGF receptors could lead to disturbances in facial and spinal bone embryogenesis. In addition, transforming growth factors is a name that refers to a group of compounds which also contains BMPs [48].

 The recent studies mostly focused on the effect of PDGF on mesenchymal stem cells (MSCs). Kreja et al. suggested that human nonresorbing osteoclasts could induce migration and osteogenic differentiation (OD) of MSCs, and effects on MSCs migration might be mainly due to PDGF-BB [49]. Ng et al. identified that activin-mediated TGF-beta signaling, PDGF signaling, and fibroblast growth factor (FGF) signaling as the key pathways involved in MSCs differentiation. Meanwhile, genes of the PDGF pathway are expressed strongly in undifferentiated MSCs. Fresh frozen pooled plasma (FFPP), which is rich in PDGF, has been used to replace serum for MSCs culture [50]. Nur77 and Nurr1 are members of NR4A nuclear orphan receptor family, and Maijenburg et al. found that their expression is rapidly increased upon exposure of fetal bone marrow MSCs (FBMSC) to the migratory stimuli stromal-derived factor-1*α* (SDF-1*α*) and platelet-derived growth factor-BB [51]. 

#### 3.2.2. Transforming Growth Factor Beta

 Among TGFs found in PRP, TGF-*β*1 and *β*2 are basic growth factors and differentiation factors which are involved in connective tissue healing and bone regeneration. TGF-*β* could activate the Smad path (Smad2 and Smad3) through the Serine/threonine kinase receptors I and II [52]. TGF-*β* has been observed to promote extracellular matrix production [53], stimulate biosynthesis of type I collagen and fibronectin, and induce deposition of bone matrix [54]. Accordingly, TGF-*β* could not only initiate bone regeneration but also support long-term healing and bone regeneration, and also remodelling of the maturing bone transplant [55, 56]. However, the most important function of TGF-*β*1 and -*β*2 is chemotaxis and mitogenesis of preosteoblasts and the ability to stimulate collagen deposition during connective tissue healing and bone formation [57]. Moreover, this factor inhibits osteoclast formation and bone resorption, which contributes to the predominance of bone formation over bone resorption [58]. And TGF-*β* could start the signal path of osteoprogenitor cell synthetizing BMP, regulating the expression of growth factors in bone and cartilage tissue [59].

#### 3.2.3. Insulin-Like Growth Factor 1

 The third important protein appearing in platelet granules in the blood is the IGF-1. IGF-1 deposits in bone matrix, endotheliocyte, and chondrocyte, releases during bone regeneration process and is responsible for the bone formation-bone resorption interaction [60]. The presence of IGF-1 in platelets could influence osteoblasts and preosteoblasts, initiate osteogenesis, and inhibit the apoptosis of the bone cells and expression of the mesenchymal collagen enzyme, decreasing its degradation [61]. Meanwhile, IGF-1 could bind to a specific receptor on the cell membrane and stimulate cells which take part in osteogenesis. The study showed that application of IGF-1 to the surface of rat molars could promote cementogenesis, and in combination with PDGF, bone formation on implant surfaces could be increased [62]. In addition, the biological effect of IGF-1 could be regulated by IGF binding proteins (IGFBPs), and IGFBP could transport the IGF-1 and increase its half-life period [63].

### 3.3. The Effect of PRP in Angiogenesis Factors Promoting Bone Reparation

 Osteogenesis needs sufficient blood supply, and in the last remolding stage of endochondral ossification, specified matrix metalloproteinase could degrade cartilage and bone to cause vessel grow. There are two independent ways of angiogenesis: one depends on VEGF, and the other depends on angiogenin. VEGF mainly affects new-born vessels growing and specific mitogen of endothelial cell, while angiogenin mainly affects large vessels and collateral circulation forming. 

 It is a vital step to promote angiogenesis rapidly in the bone graft in the early stage and long-term process of ossification. Local application of vascular growth factor (VGF) is proved advantageous for local vessels growth, skeletogenous cell aggregation and ossification, and adipose stem cell (ASC) could have some effects in this process [64]. Holstein et al. showed that the angiogenesis was extremely active in the process of bone repair in a mouse cranial defects model [65]. Some other researchers found that angiogenesis factors could promote bone repair, inversely antiangiogenesis factors could suppress it.

The sufficient VGFs in PRP and the quick mobilization of growth factors could be in favour of the local vessel growth, especially in angiogenesis of no artificial bone graft of cells. Some factors are considered to be associated with increasing the vascularization potential of PRP, including the concentration of plasmase, activation of Ca^2+^, releasing of VEGF, formation of platelet, and only containing histomonocyte in leucocyte [66]. Kim et al. used PRP (which contains sufficient VGF, VEGF, PMP, and peripheral blood mononuclear cells (PBMNCs) and no peripheral blood heterophil granulocyte (PBPMNs)) and transplanted into defective skull of rats. They found that angiogenic factor-enriched PRP could lead to faster and more extensive new bone formation in the critical size bone defect, and rapid angiogenesis in the initial healing period by PRP could be supposed as a way to overcome short-term effect of the rapid angiogenesis [10]. In addition, Annabi et al. studied a platelet-derived bioactive lysophospholipid, named S1P, and indicated a crucial role for S1P/EDG-1-mediated angiogenic and survival events in the regulation of microvascular network remodeling by MSC which might provide a new molecular link between hemostasis and angiogenesis processes [67].

 Marrow-original mesenchyme stem cells play an important role in vessel growth, especially in ischemia tissue and tumor. It is known that VEGF can aggregate MSC to new vessels and regulate MSCs differentiating to vessel cells. Steffen Massberg showed that platelets could provide the critical signal that recruits CD34+ bone marrow cells and c-Kit+ Sca-1+ Lin− bone marrow-derived progenitor cells to sites of vascular injury. Correspondingly, specific inhibition of platelet adhesion virtually abrogated the accumulation of both CD34+ and c-Kit+ Sca-1+ Lin− bone marrow-derived progenitor cells at sites of endothelial disruption. Binding of bone marrow cells to platelets involves both P-selectin and GPIIb integrin on platelets [68]. However, there has not been found any VEGF receptors on MSCs. Ball et al. found that VEGF-A could stimulate PDGS receptors and regulate the generation and transformation of MSCs, implying that VEGF-A/PDGF receptors could have an effect in aggregating MSC to ischemia region to promote vessels formation [69].

### 3.4. Other Mechanisms of PRP

 Platelet resuspension solution (PRS) is another product of platelet. Chevallier et al. found that MSCs cultured in PRS both accelerated the expansion rate over serial passages and spontaneously induces osteoblastic gene expression such as alkaline phosphatase (ALP), bone sialoprotein (BSP), osteopontin (Op), and bone morphogenetic protein-2 (BMP-2) in vitro, implying that PRS could accelerate MSC proliferation and enhance MSCs osteogenic differentiation [70]. Verrier et al. supplemented PRS in the cultures of MSCs assessed the typical osteoblastic markers at up to 28 days postconfluence, and showed an increased expression of typical osteoblastic marker genes such as collagen I alpha 1, bone sialoprotein II, BMP-2, and MMP-13, as well as increased Ca^2+^ incorporation, suggesting that the effect of PRS on human MSC could be at least partially mediated by BMP-2 [71]. But there has not been article reporting the interaction between PRP and BMP-2.

Meanwhile, Duan et al. found that PRP could stimulate initial growth of MSCs in a COX2 partially dependent manner, and this process could be depressed by Celebrex [72], implying that the mechanism of PRP might be related with a pathway of prostaglandin. But researches in this area are rare, so concrete mechanism is unknown. In addition, the contents of fibronectin, vitronectin, and fibrin in PRP are also higher than normal tissue. The fibronectin and vitronectin are important adhesion molecules in bone reparation, and fibrin plays a frame-like role in cell transformation.

The effect of platelet to promote bone repair may stay in early stage, because the life of platelet is only about 5–7 days. After 10 minutes of thrombosis, platelet microne begins releasing into the trauma circumstance, and full releases in a hour almost [33]. Although protein releasing could last an hour, the half life of GF and other cell factors just last few minutes. In the following stage, macrophage, aggregated by PDGF, might play a more important role. After PRP being injected into the defective region of bone, it could form a low oxygen acid die cavity which contains sufficient platelet, karyocyte, leucocyte, and collagen fiber, adjacent with the bone cell, osteoblast, and MSC. The oxygen difference between die cavity and the surrounding tissue could promote macrophage aggregating in trauma site.

## 4. The Application of PRP in Bone Healing

Studies of tissue engineering showed that composite tissue engineering scaffolds with growth factors have good bone inductivity and conductivity. Likewise, the application of PRP composite artificial bone or bio-derived bone showed the same conclusion. So far, a number of studies have focused on how PRP affected bone healing with or without growth factors and bone graft. 

Studies showed that the use of PRP with bone graft could significantly improve the quality of bone healing in rabbit model [73, 74]. Hakimi et al. demonstrated that PRP combined with autologous cancellous graft leads to a significantly better bone regeneration compared to isolated application of autologous cancellous graft in an in vivo critical size defect on load-bearing long bones of minipigs [75]. Meanwhile, Yamada et al. demonstrated in a canine model that the combination of mesenchymal stem cells with PRP resulted in a higher maturation of bone [76]. Similarly, Han et al. recently published an article based on the use of PRP as an autologous source of growth factors that can enhance the quality and quantity of osteogenesis [77]. The use of isolated cells with a biocompatible matrix in combination with PRP maximizes the effects of growth factors on these cells [12]. In addition, Giovanini et al. evaluated the effect of platelet-rich plasma (PRP) and autograft on the presence of type III and type I collagens, as well as the presence of CD34+ progenitor cells of the bone tissue in bone defect model on the calvarium of 23 rabbits. They found that the use of PRP in this study could hinder bone deposition, also enhanced type III to type I collagen ratio and the chemotaxis of CD34+ progenitor cells [78].

However, the studies on the effect of PRP on bone healing were not all positive. Sanchez et al. applied PRP to clinical nonunions and reported on their retrospective case after failed surgical fixation 21 months previously [79]. Mixed results were reported and therefore no definitive conclusions could be drawn. 

## 5. The Prospect of the Application of PRP in Bone Healing

The repair of large segmental bone defects due to trauma, inflammation, and tumor surgery remains a major clinical problem. Animal models were developed to test bone repair by tissue engineering approaches, mimicking real clinical situations. Studies differed with regard to animals, treated bone, chemistry, and structure of the scaffolds. As the advantage of PRP as a matrix for cells is that PRP is autologous and nontoxic, it is inherently safe, and any concerns of disease transmission such HIV, hepatitis, or Creutzfeldt-Jakob disease or immunogenic reactions that exist with allograft or xenograft preparations are eliminated. However, the preparation of PRP involves isolation of the PRP, after which gel formation is accelerated using calcium chloride and bovine thrombin. The use of bovine thrombin has been reported to be associated with the development of antibodies to factors V and XI, resulting in the risk of life-threatening coagulopathies [80]. Despite some benefits demonstrated to date, it must be acknowledged that the uses of PRP in bone healing applications are still weakly supported. Inferences regarding the potential establishment of platelet therapy as a reliable, efficacious, and safe therapy in managing the bone wound will require the completion of high-quality clinical trials with long-term followup. In particular, the supply of oxygen and nutrients to the cells in the inner part of the implanted scaffolds remains a major concern, requiring additional investigations [8].

## Figures and Tables

**Table 1:** Growth factors and cytokines in PRP in different mechanisms.

Mechanisms	Growth factors and cytokines	Function
Proinflammatory cytokines	IL1, IL6, and TNF-alpha [26, 27]	Important role in the early responses of bone repair

Growth factors	Platelet-derived growth factor (PDGF) [36], transforming growth factor (TGF)-beta, platelet-derived epidermal growth factor (PDEGF), platelet-derived angiogenesis factor (PDAF) [37], insulin-like growth factor (IGF-1), and platelet factor 4 (PF-4) [38, 39], vascular endothelial growth factors (VEGF), and endothelial growth factors (EGF) [40, 41]	Help the regeneration of tissues with low healing potential, potentially restoring biomechanical properties similar to normal bone

Angiogenesis factors	Vascular growth factor (VGF), VEGF, platelet derived membrane microparticles (PMP), and peripheral blood mononuclear cells (PBMNCs) [10]	Promote angiogenesis rapidly in the bone graft in the early stage

Factors in other mechanisms of PRP	Serotonin, histamine, dopamine, calcium, and adenosine [18]	In the dense granules in platelets and have fundamental effects on the biologic aspects of wound healing

## References

[1] Calori GM, Mazza E, Colombo M, Ripamonti C (2011). The use of bone-graft substitutes in large bone defects: any specific needs?. *Injury*.

[2] Zimmermann G, Moghaddam A (2011). Allograft bone matrix versus synthetic bone graft substitutes. *Injury*.

[3] Hak DJ (2007). The use of osteoconductive bone graft substitutes in orthopaedic trauma. *The Journal of the American Academy of Orthopaedic Surgeons*.

[4] Pieske O, Wittmann A, Zaspel J (2009). Autologous bone graft versus demineralized bone matrix in internal fixation of ununited long bones. *Journal of Trauma Management and Outcomes*.

[5] Giannoudis PV, Dinopoulos H, Tsiridis E (2005). Bone substitutes: an update. *Injury*.

[6] Williams A, Szabo RM (2004). Bone transplantation. *Orthopedics*.

[7] de Long WG, Einhorn TA, Koval K (2007). Bone grafts and bone graft substitutes in orthopaedic trauma surgery: a critical analysis. *Journal of Bone and Joint Surgery A*.

[8] Cancedda R, Giannoni P, Mastrogiacomo M (2007). A tissue engineering approach to bone repair in large animal models and in clinical practice. *Biomaterials*.

[9] Berner A, Boerckel JD, Saifzadeh S (2012). Biomimetic tubular nanofiber mesh and platelet rich plasma-mediated delivery of BMP-7 for large bone defect regeneration. *Cell and Tissue Research*.

[10] Kim ES, Kim JJ, Park EJ (2010). Angiogenic factor-enriched platelet-rich plasma enhances *in vivo* bone formation around alloplastic graft material. *The Journal of Advanced Prosthodontics*.

[11] Oryan A, Parizi AM, Shafiei-Sarvestani Z, Bigham AS (2012). Effects of combined hydroxyapatite and human platelet rich plasma on bone healing in rabbit model: radiological, macroscopical, hidtopathological and biomechanical evaluation. *Cell and Tissue Banking*.

[12] Foster TE, Puskas BL, Mandelbaum BR, Gerhardt MB, Rodeo SA (2009). Platelet-rich plasma: from basic science to clinical applications. *The American Journal of Sports Medicine*.

[13] Marx RE (2001). Platelet-rich plasma (PRP): what is PRP and what is not PRP?. *Implant Dentistry*.

[14] Anitua E, Andia I, Ardanza B, Nurden P, Nurden AT (2004). Autologous platelets as a source of proteins for healing and tissue regeneration. *Thrombosis and Haemostasis*.

[15] Ingham E, Fisher J (2005). The role of macrophages in osteolysis of total joint replacement. *Biomaterials*.

[16] Stea S, Visentin M, Granchi D (2000). Cytokines and osteolysis around total hip prostheses. *Cytokine*.

[17] Zimmerman GA, McIntyre TM, Prescott SM, Stafforini DM (2002). The platelet-activating factor signaling system and its regulators in syndromes of inflammation and thrombosis. *Critical Care Medicine*.

[18] Chesney CM, Pifer DD, Byers LW, Muirhead EE (1982). Effect of platelet-activating factor (PAF) on human platelets. *Blood*.

[19] Jurk K, Kehrel BE (2005). Platelets: physiology and biochemistry. *Seminars in Thrombosis and Hemostasis*.

[20] Glass GE, Chan JK, Freidin A, Feldmann M, Horwood NJ, Nanchahal J (2011). TNF-*α* promotes fracture repair by augmenting the recruitment and differentiation of muscle-derived stromal cells. *Proceedings of the National Academy of Sciences of the United States of America*.

[21] Kamoda H, Yamashita M, Ishikawa T (2012). Platelet-rich plasma combined with hydroxyapatite for lumbar interbody fusion promoted bone formation and decreased an inflammatory pain neuropeptide in rats. *Spine*.

[22] Zheng GH, Xiong SQ, Mei LJ, Chen HY, Wang T, Chu JF (2012). Elevated plasma platelet activating factor, platelet activating factor acetylhydrolase levels and risk of coronary heart disease or blood stasis syndrome of coronary heart disease in chinese: a case control study: a case-control study. *Inflammation*.

[23] McManus LM, Pinckard RN (2000). PAF, a putative mediator of oral inflammation. *Critical Reviews in Oral Biology and Medicine*.

[24] Mishra A, Woodall J, Vieira A (2009). Treatment of tendon and muscle using platelet-rich plasma. *Clinics in Sports Medicine*.

[25] Hashikawa T, Takedachi M, Terakura M (2003). Involvement of CD73 (ecto-5′-nucleotidase) in adenosine generation by human gingival fibroblasts. *Journal of Dental Research*.

[26] David JP, Schett G (2010). TNF and bone. *Current Directions in Autoimmunity*.

[27] Cachaço AS, Carvalho T, Santos AC (2010). TNF-*α* regulates the effects of irradiation in the mouse bone marrow microenvironment. *PLoS ONE*.

[28] Lyras DN, Kazakos K, Verettas D (2009). The influence of platelet-rich plasma on angiogenesis during the early phase of tendon healing. *Foot and Ankle International*.

[29] Lyras DN, Kazakos K, Verettas D (2009). The effect of platelet-rich plasma gel in the early phase of patellar tendon healing. *Archives of Orthopaedic and Trauma Surgery*.

[30] Murray MM, Spindler KP, Abreu E (2007). Collagen-platelet rich plasma hydrogel enhances primary repair of the porcine anterior cruciate ligament. *Journal of Orthopaedic Research*.

[31] Spindler KP, Murray MM, Carey JL, Zurakowski D, Fleming BC (2009). The use of platelets to affect functional healing of an anterior cruciate ligament (ACL) autograft in a caprine ACL reconstruction model. *Journal of Orthopaedic Research*.

[32] Taylor DW, Petrera M, Hendry M, Theodoropoulos JS (2011). A systematic review of the use of platelet-rich plasma in sports medicine as a new treatment for tendon and ligament injuries. *Clinical Journal of Sport Medicine*.

[33] Pietrzak WS, Eppley BL (2005). Platelet rich plasma: biology and new technology. *Journal of Craniofacial Surgery*.

[34] Sharma P, Maffulli N (2005). Tendon injury and tendinopathy: healing and repair. *Journal of Bone and Joint Surgery A*.

[35] Frank C, Mcdonald D, Shrive N (1997). Collagen fibril diameters in the rabbit medial collateral ligament scar: a longer term assessment. *Connective Tissue Research*.

[36] Mannaioni PF, Di Bello MG, Masini E (1997). Platelets and inflammation: role of platelet-derived growth factor, adhesion molecules and histamine. *Inflammation Research*.

[37] van den Dolder J, Mooren R, Vloon APG, Stoelinga PJW, Jansen JA (2006). Platelet-rich plasma: quantification of growth factor levels and the effect on growth and differentiation of rat bone marrow cells. *Tissue Engineering*.

[38] Sharif PS, Abdollahi M (2010). The role of platelets in bone remodeling. *Inflammation and Allergy—Drug Targets*.

[39] Bowen-Pope DF, Raines EW (2011). History of discovery: platelet-derived growth factor. *Arteriosclerosis, Thrombosis, and Vascular Biology*.

[40] Pintucci G, Froum S, Pinnell J, Mignatti P, Rafii S, Green D (2002). Trophic effects of platelets on cultured endothelial cells are mediated by platelet-associated fibroblast growth factor-2 (FGF-2) and vascular endothelial growth factor (VEGF). *Thrombosis and Haemostasis*.

[41] Salgado R, Benoy I, Bogers J (2001). Platelets and vascular endothelial growth factor (VEGF): a morphological and functional study. *Angiogenesis*.

[42] Alsousou J, Thompson M, Hulley P, Noble A, Willett K (2009). The biology of platelet-rich plasma and its application in trauma and orthopaedic surgery: a review of the literature. *Journal of Bone and Joint Surgery B*.

[43] Miyazono K, Takaku F (1989). Platelet-derived growth factors. *Blood Reviews*.

[44] Nikolidakis D, Jansen JA (2008). The biology of platelet-rich plasma and its application in oral surgery: literature review. *Tissue Engineering B*.

[45] Kao HK, Chen B, Murphy GF, Li Q, Orgill DP, Guo L (2011). Peripheral blood fibrocytes: enhancement of wound healing by cell proliferation, re-epithelialization, contraction, and angiogenesis. *Annals of Surgery*.

[46] Zhang Y, Chen J, Zhong ZM, Yang D, Zhu Q (2010). Is platelet-derived growth factor-BB expression proportional to fibrosis in the hypertrophied lumber ligamentum flavum?. *Spine*.

[47] Eppley BL, Pietrzak WS, Blanton M (2006). Platelet-rich plasma: a review of biology and applications in plastic surgery. *Plastic and Reconstructive Surgery*.

[48] Salib RJ (2007). Transforming growth factor-*β* gene expression studies in nasal mucosal biopsies in naturally occuring allergic rhinitis. *Annals of the Royal College of Surgeons of England*.

[49] Kreja L, Brenner RE, Tautzenberger A (2010). Non-resorbing osteoclasts induce migration and osteogenic differentiation of mesenchymal stem cells. *Journal of Cellular Biochemistry*.

[50] Ng F, Boucher S, Koh S (2008). PDGF, tgf-2. And FGF signaling is important for differentiation and growth of mesenchymal stem cells (mscs): transcriptional profiling can identify markers and signaling pathways important in differentiation of MSCs into adipogenic, chondrogenic, and osteogenic lineages. *Blood*.

[51] Maijenburg MW, Gilissen C, Melief SM (2012). Nuclear receptors Nur77 and Nurr1 modulate mesenchymal stromal cell migration. *Stem Cells and Development*.

[52] Heldin CH, Miyazono K, Dijke PT (1997). TGF-*β* signalling from cell membrane to nucleus through SMAD proteins. *Nature*.

[53] Wrana JL, Maeno M, Hawrylyshyn B, Yao KL, Domenicucci C, Sodek J (1988). Differential effects of transforming growth factor-*β* on the synthesis of extracellular matrix proteins by normal fetal rat calvarial bone cell populations. *Journal of Cell Biology*.

[54] Bonewald LF, Mundy GR (1990). Role of transforming growth factor-*β* in bone remodeling. *Clinical Orthopaedics and Related Research*.

[55] Hsiao ST, Asgari A, Lokmic Z (2012). Comparative analysis of paracrine factor expression in human adult mesenchymal stem cells derived from bone marrow, adipose, and dermal tissue. *Stem Cells and Development*.

[56] Weiss S, Zimmermann G, Pufe T, Varoga D, Henle P (2009). The systemic angiogenic response during bone healing. *Archives of Orthopaedic and Trauma Surgery*.

[57] Beck LS, DeGuzman L, Lee WP, Xu Y, Siegel MW, Amento EP (1993). One systemic administration of transforming growth factor-*β*1 reverses age- or glucocorticoid-impaired wound healing. *Journal of Clinical Investigation*.

[58] Wrotniak M, Bielecki T, Gaździk TS (2007). Current opinion about using the platelet-rich gel in orthopaedics and trauma surgery. *Ortopedia Traumatologia Rehabilitacja*.

[59] Panseri S, Russo A, Cunha C (2011). Osteochondral tissue engineering approaches for articular cartilage and subchondral bone regeneration. *Knee Surgery, Sports Traumatology, Arthroscopy*.

[60] Mohan S, Baylink DJ (2002). IGF-binding proteins are multifunctional and act via IGF-dependent and -independent mechanisms. *Journal of Endocrinology*.

[61] Joseph BK, Savage NW, Daley TJ, Young WG (1996). In situ hybridization evidence for a paracrine/autocrine role for insulin-like growth factor-I in tooth development. *Growth Factors*.

[62] Stefani CM, Machado MAN, Sallum EA, Sallum AW, Toledo S, Nociti FH (2000). Platelet-derived growth factor/insulin-like growth factor-1 combination and bone regeneration around implants placed into extraction sockets: a histometric study in dogs. *Implant Dentistry*.

[63] Govoni KE, Baylink DJ, Mohan S (2005). The multi-functional role of insulin-like growth factor binding proteins in bone. *Pediatric Nephrology*.

[64] Behr B, Tang C, Germann G, Longaker MT, Quarto N (2011). Locally applied vascular endothelial growth factor A increases the osteogenic healing capacity of human adipose-derived stem cells by promoting osteogenic and endothelial differentiation. *Stem Cells*.

[65] Holstein JH, Becker SC, Fiedler M (2011). Intravital microscopic studies of angiogenesis during bone defect healing in mice calvaria. *Injury*.

[66] Karp JM, Tanaka TS, Zohar R (2005). Thrombin mediated migration of osteogenic cells. *Bone*.

[67] Annabi B, Thibeault S, Lee YT (2003). Matrix metalloproteinase regulation of sphingosine-1-phosphate-induced angiogenic properties of bone marrow stromal cells. *Experimental Hematology*.

[68] Massberg S, Konrad I, Schürzinger K (2006). Platelets secrete stromal cell-derived factor 1*α* and recruit bone marrow-derived progenitor cells to arterial thrombi *in vivo*. *Journal of Experimental Medicine*.

[69] Ball SG, Shuttleworth CA, Kielty CM (2007). Mesenchymal stem cells and neovascularization: role of platelet-derived growth factor receptors: angiogenesis review series. *Journal of Cellular and Molecular Medicine*.

[70] Chevallier N, Anagnostou F, Zilber S (2010). Osteoblastic differentiation of human mesenchymal stem cells with platelet lysate. *Biomaterials*.

[71] Verrier S, Meury TR, Kupcsik L, Heini P, Stoll T, Alini M (2010). Platelet-released supernatant induces osteoblastic differentiation of human mesenchymal stem cells: potential role of BMP-2. *European Cells and Materials*.

[72] Duan J, Kuang W, Tan J (2011). Differential effects of platelet rich plasma and washed platelets on the proliferation of mouse MSC cells. *Molecular Biology Reports*.

[73] Kanthan SR, Kavitha G, Addi S, Choon DSK, Kamarul T (2011). Platelet-rich plasma (PRP) enhances bone healing in non-united critical-sized defects: a preliminary study involving rabbit models. *Injury*.

[74] Zhang YD, Wang G, Sun Y, Zhang CQ (2011). Combination of platelet-rich plasma with degradable bioactive borate glass for segmental bone defect repair. *Acta Orthopaedica Belgica*.

[75] Hakimi M, Jungbluth P, Sager M (2010). Combined use of platelet-rich plasma and autologous bone grafts in the treatment of long bone defects in mini-pigs. *Injury*.

[76] Yamada Y, Ueda M, Naiki T, Takahashi M, Hata KI, Nagasaka T (2004). Autogenous injectable bone for regeneration with mesenchymal stem cells and platelet-rich plasma: tissue-engineered bone regeneration. *Tissue Engineering*.

[77] Han B, Woodell-May J, Ponticiello M, Yang Z, Nimni M (2009). The effect of thrombin activation of platelet-rich plasma on demineralized bone matrix osteoinductivity. *Journal of Bone and Joint Surgery A*.

[78] Giovanini AF, Deliberador TM, Gonzaga CC (2010). Platelet-rich plasma diminishes calvarial bone repair associated with alterations in collagen matrix composition and elevated CD34+ cell prevalence. *Bone*.

[79] Sanchez M, Anitua E, Cugat R (2009). Nonunions treated with autologous preparation rich in growth factors. *Journal of Orthopaedic Trauma*.

[80] Sánchez AR, Sheridan PJ, Kupp LI (2003). Is platelet-rich plasma the perfect enhancement factor? A current review. *International Journal of Oral and Maxillofacial Implants*.

